# Interphotoreceptor coupling: an evolutionary perspective

**DOI:** 10.1007/s00424-021-02572-9

**Published:** 2021-05-14

**Authors:** Lorenzo Cangiano, Sabrina Asteriti

**Affiliations:** Dept. of Translational Research, University of Pisa, Via San Zeno 31, 56123 Pisa, Italy

**Keywords:** Gap junctions, Photoreceptors, Rods, Cones, Coupling, Evolution

## Abstract

In the vertebrate retina, signals generated by cones of different spectral preference and by highly sensitive rod photoreceptors interact at various levels to extract salient visual information. The first opportunity for such interaction is offered by electrical coupling of the photoreceptors themselves, which is mediated by gap junctions located at the contact points of specialised cellular processes: synaptic terminals, telodendria and radial fins. Here, we examine the evolutionary pressures for and against interphotoreceptor coupling, which are likely to have shaped how coupling is deployed in different species. The impact of coupling on signal to noise ratio, spatial acuity, contrast sensitivity, absolute and increment threshold, retinal signal flow and colour discrimination is discussed while emphasising available data from a variety of vertebrate models spanning from lampreys to primates. We highlight the many gaps in our knowledge, persisting discrepancies in the literature, as well as some major unanswered questions on the actual extent and physiological role of cone-cone, rod-cone and rod-rod communication. Lastly, we point toward limited but intriguing evidence suggestive of the ancestral form of coupling among ciliary photoreceptors.

## Introduction

While the focus of this review is on interphotoreceptor electrical coupling, the reader should be aware that gap junctions occur throughout the retina, between neurons both of the same and of different types (for review see [129]). In some cases, their physiological function is well established, as that between AII amacrines in the so-called *primary rod pathway*, which is crucial for mammalian night vision ([52, 85] and references therein). In other cases, much less is known, and more subtle and complex roles are progressively being untangled ([117] and references therein). Also, beyond the scope of this review is a discussion of the involvement of gap junctions in the development of retinal circuits. Here, we examine the anatomical substrates and possible physiological roles of interphotoreceptor coupling in the adult retina. As we shall see below, the pressures for or against interphotoreceptor coupling throughout vertebrate evolution are likely to have varied greatly depending on the identity of the partners: cone-cone, rod-cone, rod-rod. It is thus important to briefly examine the intricate evolutionary history of cones and rods, as differences in interphotoreceptor coupling between species may, to a large extent, reflect their specific route from our early vertebrate ancestors.

## The complex history of cone and rod photoreceptors

The relative abundance of cones and rods varies considerably in different vertebrate retinas. Examples at the opposite ends of the spectrum are the cone-dominated retinas of diurnal lizards [16] and the pure rod retinas of several skates and deep sea teleost fishes [105, 130]. However, in many such extremes of over/under-representation, the photoreceptors have been found to display a complex mixture of morphological and functional properties that prevent their simple categorisation as cones or rods. For instance, skate rods can slowly adapt to bright light, that is to luminance levels normally handled by cones in duplex retinas [105]. In fact, a growing body of evidence from reptiles and teleosts supports the occurrence of multiple independent events of partial ‘transmutation’ of cones into rods and vice versa (see [17, 21]), an idea originally proposed by Walls in 1942 [131] (for a modern view of the different possible modes of photoreceptor evolution see [89]). These observations demonstrate a major retuning of photoreceptor phenotypes in response to the prior loss of rods or cones [111], or accompanying highly specialised ecological adaptations such as foraging in mesopic ambient luminance [21, 29]. While the occurrence of these phenomena throughout vertebrate evolutionary history complicates any effort to rigorously define a rod and a cone, there is a broadly recurring pattern across distant species whereby dim light vision is handled by neurons expressing an Rh1 pigment and a similar set of rod-like phototransduction cascade enzymes [72]. Such evidence continues to support the original proposal by Schultze [112], later formulated as the ‘duplicity theory’ (reviewed by [131]), of a division of roles between rods and cones as a general vertebrate *bauplan*. It is now widely accepted that such highly sensitive ‘rod’ photoreceptor originally evolved from an ancestral cone (reviewed in [71, 73]). Recent electrophysiological data from lampreys, our most distant extant relatives whose line diverged from other vertebrates more than 500 million years ago, suggests that a rod-like photoreceptor was already present in our last common ancestor in the Cambrian period [7, 50, 88, 107]. In fact, molecular phylogenetic analysis points to the rod-cone divergence occurring much before the vertebrate radiation [72].

Moving forward in evolution, with a few exceptions mammalian retinas are dominated by rods, even in diurnal species [98]. This phenomenon led Walls [131] to propose what is now commonly referred to as the *nocturnal bottleneck* hypothesis [53], the idea that prior to the great Cretaceous-Paleogene mass extinction event of ~ 65.5 million years ago [116], which wiped out large predatory reptiles, the precursors of modern mammals occupied a nocturnal ecological niche. Notably, evidence of a nocturnal evolutionary legacy has also been found in the mammalian eye optics [53]. Recent data from mouse suggest that the adaptation to a nocturnal visual landscape of proto-mammals may have involved the developmental recruitment of blue (S-) cone progenitors into a rod fate via activation of the transcription factor NRL [67]. In our view, this does not appear to be a case of transmutation with the meaning given in the previous paragraph but of the mere enhancement of a pre-existing ancestral regulatory pathway, since activation of the same transcription factor in zebrafish is sufficient to divert developing cones into rods [94]. Interestingly, an *Nrl* homolog is present in the lamprey genome, raising the possibility that this transcription factor already played a role in the developmental specification of a rod-like photoreceptor in our earliest vertebrate ancestors (see Sect. 14.2 in [71]). Returning to the nocturnal bottleneck, the subsequent radiation of mammals and occupation of new daytime habitats would have led to a limited increase in the developmental recruitment of cone photoreceptors, thereby explaining the relative dearth of cones in most mammalian retinas [98]. Specific mammalian adaptations go beyond photoreceptors and include neurons downstream of rods. For instance, rod bipolar cells do not contact ganglion cells directly but feed into the cone pathway via AII amacrines ([85] and references therein). Other relevant evolutionary changes will be discussed in later sections.

## The anatomical substrate of interphotoreceptor coupling

Possibly the first observations of diffuse “synaptic contacts” between photoreceptors were made by Sjöstrand [118] in guinea pig. Through ultrastructural (i.e. electron microscopic) imaging of serial sections in the outer plexiform layer he identified thin processes extending from the conical synaptic boutons of what he referred to as β-cells to the spherical boutons of α-cells: at the time the guinea pig was thought to have a pure rod retina and these terms differentiated between two forms of the rod photoreceptor. However, later work unambiguously demonstrated an abundance of cones and dichromatic vision in this species [98], implying that Sjöstrand had most likely observed telodendria extending from cone pedicles to rod spherules. Following his study, ultrastructural evidence indicative of direct cone-cone, rod-cone and rod-rod communication, emerged in a variety of species, generally model systems of broad interest for research into retinal function. Contact points were found along the entire length of the photoreceptors, except for the outer segment, frequently (but not exclusively) occurring at the tips of specialised cellular processes with striking morphologies: axonal *telodendria* and inner segment *radial fins*.

**Fig. 1:**
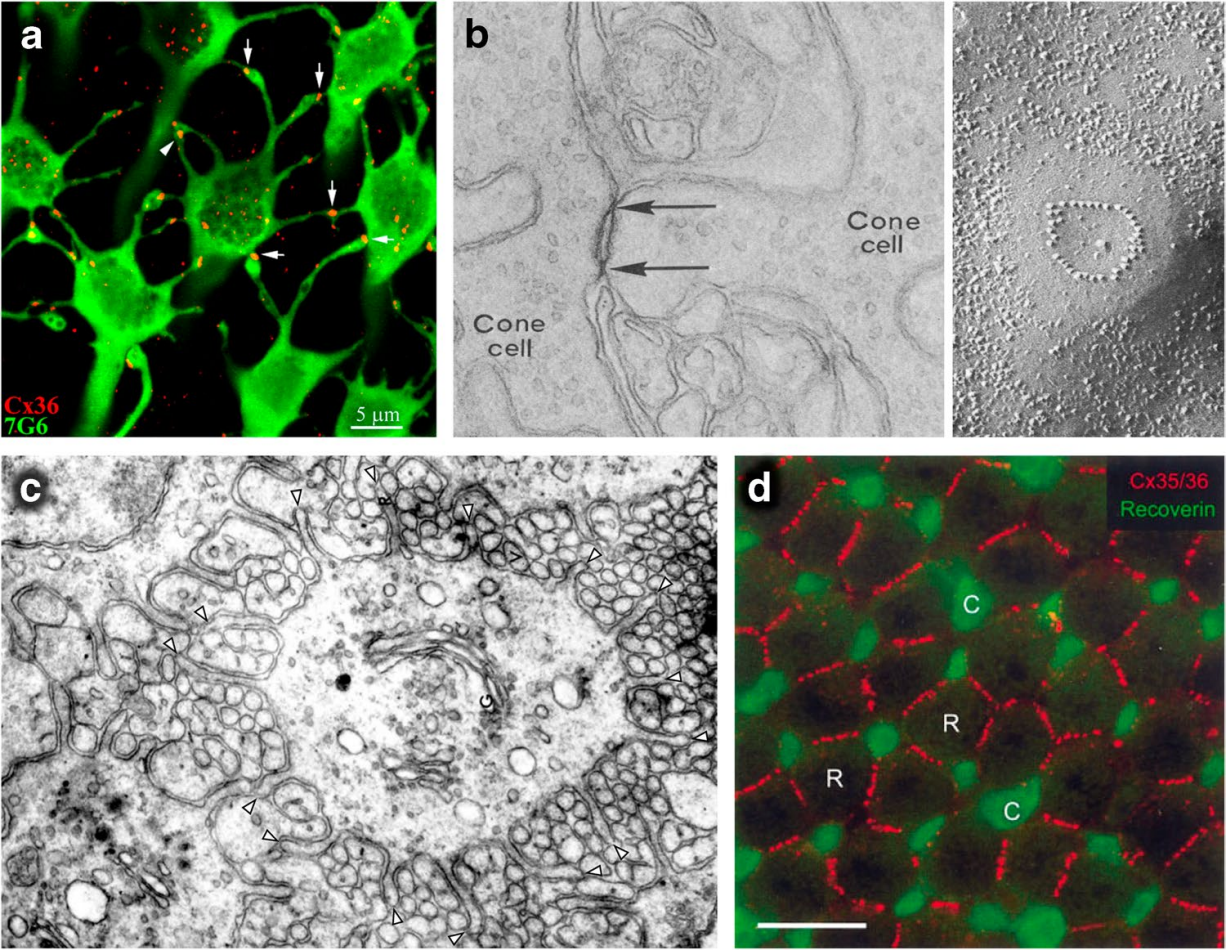
Sites of interphotoreceptor coupling. **a** Telodendria (green processes) connect adjacent cone pedicles (green polygons) in the peripheral retina of macaque and Cx36 (red) colocalises with contact points (from [93]). **b** Ultrastructure or cone-cone pedicle gap junctions in macaque seen in cross section with TEM at × 55,000 (left) and face-on in freeze fracture at × 88,000 (right) (from [100]). **c** Gear-like radial fins between pigeon photoreceptor inner segments viewed with TEM at × 35,000 (reproduced with permission from [26]). White arrowheads point to their tips. **d** Cx35 (red) colocalises with radial fins around the perimeter of salamander rod inner segments; scale bar 20 μm, R: rods, C: cones (reproduced with permission from [140])

Telodendria, known since the early anatomical work of the nineteenth century (references in [125]), are thin and long tubular processes (~ 0.1 μm in diameter) emanating from the photoreceptor axon terminal (pedicle for cones, spherule for rods) and projecting laterally in the outer plexiform layer for up to a few tens of μm (Fig. 1a). These are ubiquitous across vertebrates, having been reported or characterised in guinea pig [118], pigeon [26], human, macaque, squirrel [1, 27, 93], dogfish [120], turtle [75], salamander [33], cat [68], mouse [126], lamprey [7] and zebrafish [92]. Interestingly, while in lower vertebrates both rods and cones possess telodendria, in mammals they have been found only in cones.

Radial fins are relatively short sheet-like processes (~ 0.1 μm thick), which protrude laterally from the inner segment of both types of photoreceptors, just beyond the inner limiting membrane. They interdigitate with the fins of neighbouring neurons in an aesthetically appealing gear-like fashion (Fig. 1c) and seem to be almost as common as telodendria, having been described in lizard, turtle, finch, pigeon ([26] and references therein), lamprey [137], dogfish [120], salamander [33], toad [48], snake [54] and frog [70]. In macaque, they are also well developed, except among cones in the most central rod-less region of the retina, the foveola [19]. Their presence in the human retina is unclear [104].

In thin serial sections, both telodendria and radial fin contacts often displayed a narrowing of the intercellular cleft with an increased electron density (Fig. 1b left). A debate ensued over whether these were sites of electrical coupling rather than of intercellular mechanical anchoring [27, 33, 75, 120] and early electrophysiological evidence of coupling in turtle cones [14] did not clarify whether communication was mediated by gap junctions or neurotransmitter release. However, face-on imaging of freeze fractured retinal tissue revealed that in many species the electron dense contacts contain small particles, frequently arranged in rows, typical of gap junctions (Fig. 1b right). For axon terminals and telodendria this was confirmed in macaque, rabbit, turtle [100] and human [101], while for radial fins in toad [48] and snake [54].

The subsequent discovery that the homologous connexin isoforms 35/36 (Cx35/Cx36) are expressed in the photoreceptors of lower vertebrates and mammals, enabled to confirm that gap junctional coupling is indeed localised to telodendria in guinea pig [78], zebrafish [79], macaque (Fig. 1a) [93], human [63] and mouse [18, 80], among other species, as well as between radial fins in salamander (Fig. 1d) [140]. It must be noted that for unknown reasons, antibodies against Cx36 do not label mammalian rod-rod contacts [18, 93]. A radical explanation for this observation, recently put forward in mouse, is that rod-rod coupling may, in fact, be entirely absent [61]. Further important evidence that interphotoreceptor coupling is mediated by gap junctions comes from the use of pharmacological blockers (albeit poorly specific), which prevent the intercellular diffusion of injected dyes [56, 57] and abolish both electrically [56] and light-evoked signals [5]. Furthermore, in Cx36 knockout mice rod-cone coupling is undetectable [6, 59, 61, 124]. In spite of these data, there is sparse but intriguing evidence in the literature of vesicular neurotransmitter release between photoreceptors, which we will examine at the end of this review.

## Evolutionary pressures for and against interphotoreceptor coupling

Here, we examine the evolutionary advantages and disadvantages of coupling, emphasising, where relevant, the differing points of view of rods and cones. Such differences arise from their respective tasks in the vertebrate duplex retina: rod pathways must maximise the downstream impact of the photoreceptors’ high sensitivity, even at the cost of a loss in spatial discrimination; cone pathways, ideally should preserve spatial and, in most species, chromatic information.

### Impact on signal to noise ratio, acuity, contrast sensitivity and increment threshold

**Fig. 2:**
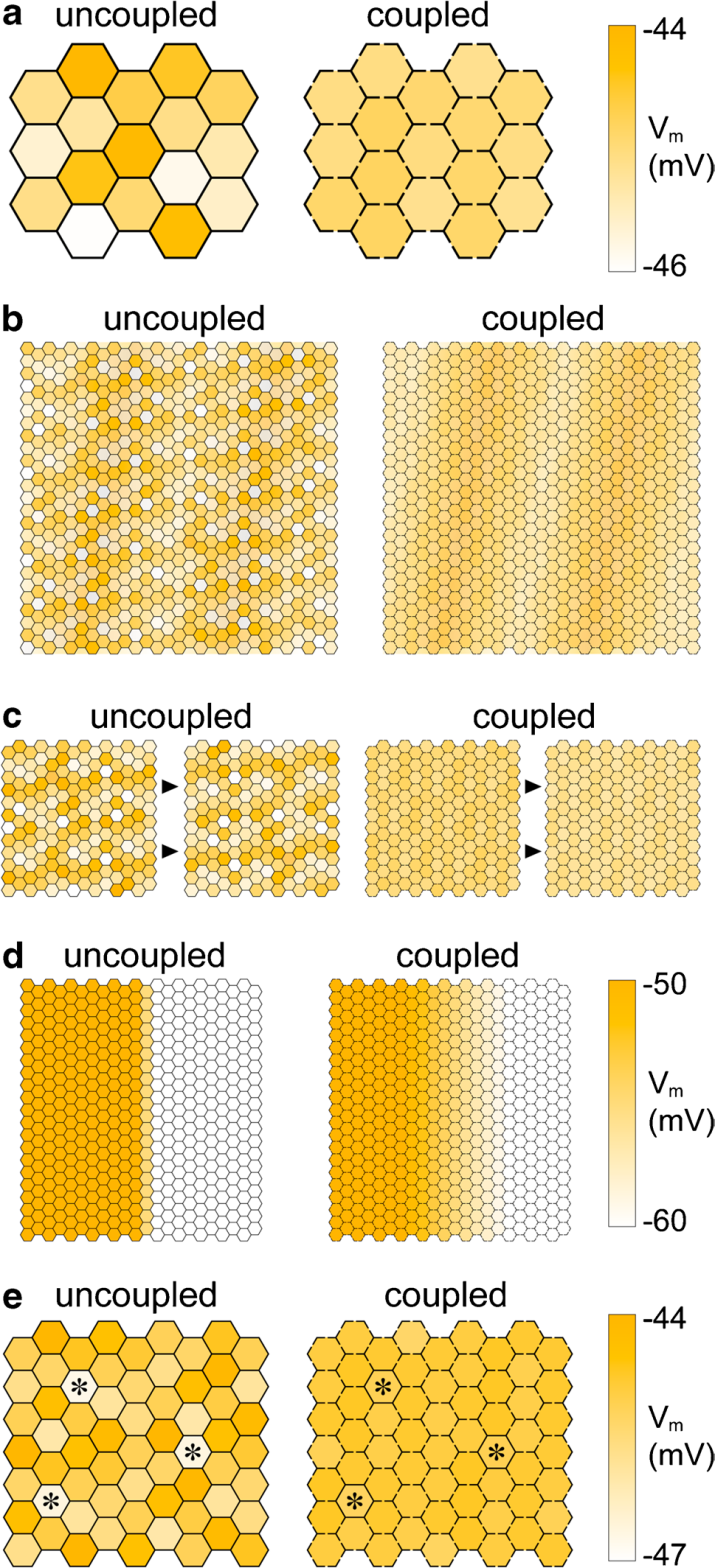
Schematic representation of the impact of coupling on visual coding by photoreceptors. **a** Various noise sources generate relatively large uncorrelated membrane potential fluctuations in uncoupled photoreceptors (left). Electrical coupling leads to an attenuation of these fluctuations through lateral averaging (right). The hexagon matrix stands for a generic rod or cone mosaic in darkness, while the colours represent a time snapshot of their membrane potentials. **b** Coupling should improve the contrast sensitivity for stimuli of low spatial frequency, as in the case shown here of diagonally-oriented very dim light bands. **c** Coupling might reduce the increment threshold of rod or cone vision. In this example, dim light is suddenly projected onto the retina. When the photoreceptors are coupled the stimulus is more easily discernible. Arrowheads indicate the flow of time. **d** Lateral averaging caused by electrical coupling has the potential to reduce spatial acuity. Here, a sharp bright edge is coded as a gradation of membrane potentials. **e** Bona fide single photon responses are difficult or impossible to recognise when rods are coupled in a syncytium (right), while this is frequently possible in uncoupled rods (left). The panel represents a population of rods in darkness, among which three photoisomerisations have just occurred (asterisks)

Seen from one photoreceptor, electrical coupling to its neighbours, irrespective of their type, has the effect of diluting its own photocurrent over a larger membrane area. With patterned light stimulation of a small portion of the retina, coupling causes an averaging of the responses of nearby photoreceptors (i.e. lateral averaging), degrading information on the spatial distribution of the stimulus [11]. What are then the potential benefits of coupling? A well-established concept is that it improves the signal to noise ratio (SNR) at the level of the photoreceptors by reducing the uncorrelated noise generated in the coupled cells [47] (Fig. 2a). Evidence in support of this effect was first obtained in turtle cones, whose membrane potential fluctuations in darkness (dark noise) were found to inversely correlate with their degree of coupling to other cones as quantified by a length constant [74]. Several are the sources of noise in photoreceptors and their respective contribution varies greatly between rods and cones [71, 90]: (i) in darkness and dim light the phototransductive cascade is intrinsically noisy, mainly due to spontaneous activation of the key enzyme cGMP phosphodiesterase; (ii) spontaneous thermal activation of the visual pigment generates a random train of spurious single photon responses (while in rods their mean interval is on the order of tens of seconds, in L-cones it is so small to dominate noise [103]); (iii) in dim light, photons rain onto the retina and are absorbed stochastically by any given outer segment; on one level this process can be considered as noise superimposed on an ideal continuous signal [39], that is the luminance of the object being observed. Cone-cone and rod-rod coupling are very effective at attenuating these uncorrelated sources of noise, roughly according to the square root of the number of coupled cells [74]. For diffuse light stimuli that coactivate all coupled photoreceptors, the SNR thus improves with the same progression. A higher SNR should improve visual system performance by increasing contrast sensitivity for patterned stimuli containing low spatial frequencies (Fig. 2b) [38, 77] and reducing *increment threshold* for both rod and cone vision (Fig. 2c). Consistent with these expected benefits, cone-cone coupling has been detected in the majority of species examined to date, with the exception of salamander cones and, possibly, mammalian S-cones. Interestingly, macaque S-cones have been found to be noisier than M/L-cones in patch recordings [13], a difference that could be linked to the weak electrical coupling of the former.

Let’s examine the potential cost imposed by lateral averaging on visual acuity (Fig. 2d), as this would likely constitute an evolutionary upper limit to coupling. The issue has been a longstanding concern in the field, as testified by a comment in [74] that their recordings were made in the peripheral turtle retina, where spatial information is likely to be of lesser importance. A crucial point to bear in mind is that the actual cost of coupling is mitigated by limitations in the eye’s optics and scattering in the retina: the in vivo image at the level of the photoreceptor outer segments of a point source in visual space (the *point spread function* of the system) has a quasi-Gaussian distribution. Measurements indicate that the half width of this distribution is comparable or exceeds the distance of adjacent photoreceptors (references in [38, 82]). In turtle, optical blurring just from scattering in the retina has been estimated to attenuate the negative impact of its tightly coupled rod syncytium on visual acuity [31, 113]. In human important information came, unexpectedly, from an elegant psychophysical experiment in which laser interferometry was used to bypass blurring by the eye’s optics and project a high spatial frequency grating on the central retina [134]. Subjects experienced aliasing (artefactual low frequency patterns) when the grating frequency exceeded the Nyquist limit calculated from cone spacing in the region of highest acuity, the rod-less foveola. Notably, aliasing does not occur in normal vision. This indicates that physiological optical blurring effectively removes any details in the projected image at the scale of the cone-cone distance, even in the foveola where thinning of the retina reduces scattering. It thus seems plausible that a moderate amount of nearest-neighbour cone-cone coupling would suppress noise without impacting acuity [38]. In fact, cones in the human and macaque foveola contact each other via a profusion of telodendrial processes [1, 125]. The question is whether these telodendria harbour functional gap junctions.

In principle, a comparable improvement in the SNR at the same cost in terms of spatial acuity could be obtained by replacing interphotoreceptor coupling with a large convergence onto bipolar cells via low gain linear synapses [12, 74]. Based on the low level of rod-rod coupling in mammals one might expect that they adopted this alternative solution. On the contrary in the mouse retina, for which we have the most reliable data, the synapse between rods and rod bipolar cells is markedly nonlinear. We discuss this conundrum in the next section.

### Impact on SNR and absolute threshold

One major downside of coupling is that, for very localised stimuli, it actually degrades the SNR approximately with the square root of the number of coupled photoreceptors. This is particularly problematic in darkness for lower vertebrates, whose rods form a tightly interconnected syncytium: while single photons evoke a relatively large decrease in the circulating current (in percentage and relative to noise, data summarised in [7]), the low input resistance of coupled photoreceptors greatly attenuates the change in membrane potential (~ 26-fold in turtle relative to hypothetically uncoupled rods [31]). The effect is so dramatic to prevent the identification of single photon responses in photovoltage records amidst biological and instrumental noise in both rods [7, 40] (Fig. 2e) and bipolar cells [3]. Lower vertebrates recover this attenuation via a high synaptic gain and spatial pooling [3, 9, 24, 30].

The absolute visual threshold of mammals corresponds to the temporal coincidence of only a few absorbed photons in a pool of thousands of rods ([90] and references therein), surprisingly similar to that of lower vertebrates ([25] and references therein). However, mammals adopted, possibly due to the nocturnal bottleneck, a particular network strategy involving two key changes (see discussion in [2]): (i) thresholding nonlinearities in rod-rod bipolar-AII amacrine cell synapses to reject dark noise and the smallest single photon responses [43]; (ii) a dramatic reduction in rod-rod coupling, reflected in small junctional contacts and patchy rod syncytia that seem to leave some rods completely uncoupled (mouse: [126]; macaque: [56]; squirrel: [82]). Recent data from mouse suggest that rods may, in fact, not couple to each other at all [61], although this must be reconciled with clear ultrastructural evidence of rod-rod gap junctions [128]. The consequence is that in mammals, single photon responses can generally be easily distinguished from noise in photovoltage records in both rods (macaque: [56]; squirrel: [82]; mouse: [22]) and rod bipolars (e.g. [23, 43]). Combined with convergence in the primary rod pathway this leads to a large increase in sensitivity and decrease in perceptual threshold in AII amacrines and ganglion cells relative to rods [39, 43].

**Fig. 3:**
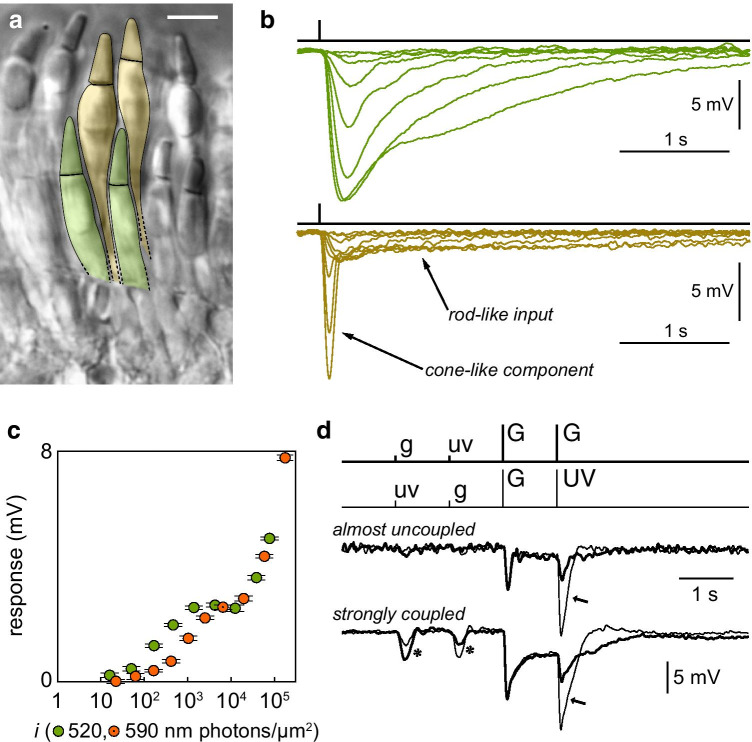
Rod-cone coupling, from lamprey to mouse. **a** Rod-like (green) and cone-like (yellow) photoreceptors of the river lamprey viewed in a retinal slice; scale bar 10 μm. **b** Membrane potential responses to flashes of light of a rod-like (top) and cone-like photoreceptor (bottom); the latter displays both slow and highly sensitive rod-like input, and its own fast response. **c** Response amplitude vs. flash strength of another lamprey cone-like photoreceptor: the biphasic trend is the combination of the highly sensitive green-preferring component from coupled rod-like cells, with its own poorly sensitive yellow-preferring component (panels a-c from [7]). **d** Photovoltage responses of a mouse mixed S/M-cone to a sequence of dim green (g, 16.6 photons/μm^2^) and ultraviolet flashes (uv, 16.6 photons/μm^2^) followed by rod-saturating bright green (G, 3140 photons/μm^2^) and ultraviolet ones (UV, 3140 photons/μm^2^). The upper pair of traces was obtained with the cone weakly coupled to rods, whereas the bottom pair was recorded a few tens of minutes later with the same cone in a much stronger state of coupling. While the intrinsic response of the cone is dominated by S-opsin as revealed after a rod-saturating preflash (arrows), coupling increases the cone’s sensitivity and shifts its spectral preference toward that of rhodopsin (asterisks) (reproduced with permission from [6])

Despite the limited amount of rod-rod coupling, mammals did retain a significant degree of rod-cone coupling, to the point of being able to confer a rod-like phenotype to coupled cones in darkness (Fig. 3d) ([5] and references below). Given the presence of a thresholding nonlinearity at the first rod synapse even modest rod-rod and rod-cone coupling are expected to have a detrimental effect on absolute visual threshold [56, 82]. This was first argued for the cat on the basis of anatomical data [119], leading the authors to propose that coupling may be dynamically regulated in response to changes in ambient light (i.e. established in mesopic conditions but suppressed in darkness). Despite this prediction, a significant degree of rod-cone coupling has been found in the fully dark-adapted retina of macaque [56] and mouse [5, 61]. Furthermore, and counterintuitively, coupling is believed to decrease with increasing light levels (we discuss modulation in a later section). The perceptual penalty of rod-rod and rod-cone coupling in mammalian scotopic vision is unknown. Li et al. [82] suggested a specific task in which some coupling would be beneficial: detection of a point light source sufficiently bright to deliver more than one photon to each of a few illuminated rods. In this case the penalty imposed by coupling on the SNR in rods could, under certain assumptions, be more than compensated for by an increase in the excitation of rod bipolar cells via recruitment of additional non saturated synapses (theoretically explored in [121]). However, it is not clear to us whether such a visual task is sufficiently relevant in the night time visual landscape to justify the accompanying degradation in absolute threshold for diffuse stimuli. If, as beetles do [34], mammals orient in moonless nights following the stars and the milky way, coupling might improve detection of relatively dim stars but deteriorate that of the diffuse milky way. Note that, from a conceptual point of view, the mechanism just mentioned falls among those discussed in the next section.

In analogy to rods, cone-cone coupling (and possibly cone-rod) is likely to affect cone absolute visual threshold. The latter was behaviourally determined in mice to correspond to ~ 5 photoisomerisations/cone for diffuse stimuli, i.e. ~ 3.5 log_10_ units higher than rod absolute threshold, but low nonetheless considering the extremely high levels of dark noise in cones [90]. The authors suggested that some form of lateral averaging might explain such perceptual performance. Clearly, cone-cone coupling is a potential candidate.

### Providing an escape route for rod or cone signals

Rod-cone coupling seems to be extremely common, having been confirmed electrophysiologically in a variety of animals, including cat [91], salamander [10], frog [70], macaque [56, 110], goldfish [102] and mouse [5]. Recently, our laboratory has found that it is present also in lamprey, where many cone-like photoreceptors receive enough rod-like photoreceptor input to acquire dim light sensitivity (Fig. 3a–c) [7]. In fact, the similarities between mouse and lamprey are striking (Fig. 3). This suggests that rod-cone coupling may have been already present in early vertebrates, perhaps since the original transmutation of an ancestral cone into the vertebrate rod. A flow of signals from rods to cones may be advantageous for visual processing when the overall gains of the indirect cone-mediated pathways are higher than the direct ones via bipolar or horizontal cells [8]. Some evidence in this direction came initially from turtle [109] and later from salamander, where it was shown that rod-horizontal cell synapses have a high gain in darkness, but saturate as soon as rods hyperpolarise by a few mV. It was thus suggested that rod-cone coupling could provide an escape route for rod signals from dim light levels upwards [9]. It seems, however, problematic that the large majority of salamander rods were found to be weakly coupled to cones in darkness [136] and that coupling increased only when light levels were sufficiently high to saturate the rods themselves [138]. In fact, the authors suggested a physiological role for the opposite pathway in brighter light, from cones to rods (rather than from rods to cones). Similar results and conclusions were later reached also in frog [70]. In striking contrast to these amphibians, rod-cone coupling in goldfish is strong at night and weak during the day [102].

**Fig. 4:**
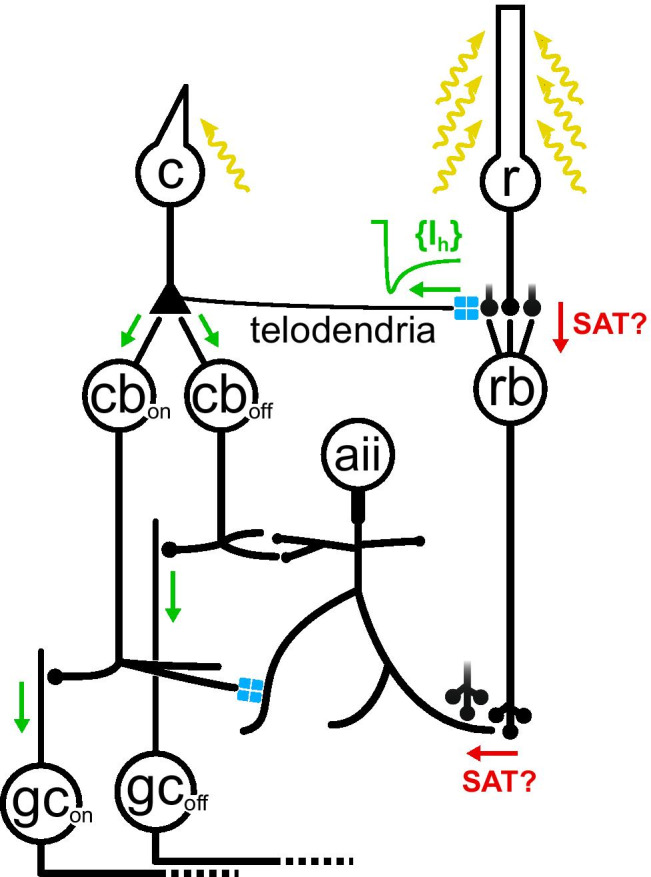
The ‘escape route’ hypothesis in the mammalian retina. When ambient light is sufficiently high to saturate the highly convergent primary rod pathway (rods➞rod bipolar cells➞AII amacrine cells➞ON and OFF cone bipolars➞ON and OFF ganglion cells), rod signals would be able to reach the inner retina via rod-cone gap junctions. In even brighter mesopic conditions, when cones generate their own intrinsic signals and rods are strongly activated, the I_h_ current would attenuate the tonic hyperpolarisation in rods and their coupled cones, thereby preventing saturation in the cone pathways. r, rods; c, cones; rb/cb, rod/cone bipolar cells; aii, AII amacrine cells; gc, ganglion cells; blue contacts, gap junctions; SAT, postulated sites of synaptic saturation

An issue highly relevant to the ‘escape route’ hypothesis is whether rod synapses that saturate as soon as these photoreceptors hyperpolarise by a few mV due to ambient light, are common among lower vertebrates and there is evidence against this ([135] and discussion therein). It must be noted that the overall gain of a particular pathway depends on convergence, noise sources, synaptic properties and adaptation at each retinal stage up to the ganglion cells [39, 43]: unfortunately, our understanding of the inner retinal processing in lower vertebrates is still rudimentary [71]. In contrast, the primary rod pathway of mammals (rods➞rod bipolars➞AII amacrines➞cone bipolars➞ganglion cells) has been thoroughly investigated ([85] and references therein). Due to its high convergence, for long it was thought to be saturated by even dim background light. This provided a logical explanation for the presence of rod-cone coupling as an escape *secondary route* for rod signals in upper scotopic and mesopic conditions (Fig. 4) (references in [52]). However, later experiments revealed that the primary route rapidly adapts and continues to operate in moderate ambient light [39, 52, 65]. Furthermore, despite evidence that rod signals reach the inner retina via gap junctions with cones (see the references just above), alternative routes have now emerged. We defer a discussion of the important issue of rod-cone coupling in mammals to the section titled ‘The perceptual impact of coupling’. Overall, rod-cone coupling may have evolved as one of several network strategies to achieve an optimal balance of rod and cone signal flow toward the inner retina, in the face of daily changes in ambient light.

### Interaction with the slow voltage–dependent conductances in the inner segment

Both rods and cones express in their inner segments voltage-dependent conductances that gate with slow kinetics (i.e. time constants from tens to hundreds of ms) and play an important role in shaping the final light response of the photoreceptor [32, 35, 37, 42, 60]. One is represented by hyperpolarisation-activated cyclic nucleotide-gated HCN1 channels (*I*_*h*_ current) and another by potassium selective channels opened by depolarisation (*I*_kx_ current; [46] and references therein). *I*_*h*_, of which more is known, is robustly expressed in all vertebrate photoreceptors including the rod-like and cone-like cells of the lamprey [7]. Both conductances antagonise changes in the photocurrent and any synaptic input to the photoreceptor in a process of slow negative feedback, conferring a band-pass behaviour to the neuron (in conjunction with membrane capacitance) ([35] and references therein). Early on this band-pass filtering was found to shape the lateral propagation of signals in the rod syncytium of turtle and salamander, whereby they become progressively more transient as they spread from cell to cell [10, 37]. The authors noted that this effect should enhance the detection of sudden changes in illumination in scotopic conditions, by collecting transient information from a large pool of coupled rods. On the contrary, for slow changes the same inner segment conductances would partially counteract the presence of coupling, thereby attenuating its degradation of spatial resolution [8, 12]. The latter property should also improve, in the presence of coupling, the encoding in the photoreceptor photovoltages of slow moving bright edges (implicit in Fig. 14A top left panel in [12]). It must be noted that mammalian rods show strong expression of the same membrane conductances despite being dramatically less mutually coupled than lower vertebrate rods, which raises doubts over the existence of a tight functional evolutionary link between rod-rod coupling and inner segment conductances.

An entirely different mechanism of interaction has been proposed for the *I*_*h*_ current and rod-cone coupling in mouse, specific to mesopic vision. This emerged from the observation that cone signalling in mesopic conditions is hampered in mice lacking the HCN1 isoform [114]. Since HCN1 channels antagonise the rod hyperpolarisation in continuous bright light (see references above), they should also act to reduce the hyperpolarisation induced in coupled cones via gap junctions (here referred to as a ‘rod offset’). As a result, synaptic saturation would be prevented in the cone ON and OFF pathways (Fig. 4). The same authors found that in HCN1-KO mice the rod offset also enters cone bipolars via AII amacrines (i.e. via the primary rod pathway). This observation leaves a degree of uncertainty over how much rod-cone coupling contributes to the deficit in mesopic vision displayed by the mutants.

### Impact on colour discrimination

Most vertebrate species retain the genes of two or more cone opsins originating from a complex history of whole and local genome duplication events ([71–73] for review). Their expression is generally (but not always) segregated in different cells, thus conferring spectral specificity to each cone with the frequent contribution of a specialised pigmented organelle in the inner segment: the oil droplet or the ellipsoid [123]. Colour discrimination requires the downstream analysis of the joint activation of these cones in what is a classical example of population coding in sensory systems. Clearly, mixing the signals generated by cones of different spectral type via gap junctions prior to the decoding step should be detrimental to colour discrimination [57, 58] and, therefore, disfavoured in evolution. This constraint is likely to have been already present in our last common ancestor with lampreys, since some species of this agnatan (jawless vertebrate) possess the substrate for sophisticated colour discrimination [133]. An animal in which much effort has been dedicated to examine whether cone-cone coupling adheres to this expectation is the turtle. While the ultrastructural data would suggest unspecific coupling, electrophysiological recordings consistently found that only cones of the same spectral type are coupled (references in [51, 92]). Similar conclusions have now been reached for the zebrafish retina (Yoshimatsu et al., bioRxiv, https://doi.org/10.1101/2020.10.26.356089). In stark contrast to the turtle and against expectations given the importance of red/green chromatic opponency for primate ethology, M- and L-cones in the peripheral macaque retina couple indiscriminately to each other [57]. A speculative hypothesis made by these authors is that this form of coupling, here referred to as *heterochromatic*, could be a transitory byproduct of the relatively recent evolutionary divergence of primate M- and L-cones from a common ancestor. If so, one would expect the much more evolutionarily distant S-cones [13] to be uncoupled from M/L-cones in all mammals. This is indeed the case in the dichromatic squirrel retina [83]. In primates, there is some occurrence of gap junctions between S- and M/L-cones [69, 93], with preliminary electrophysiological evidence suggesting that such coupling may not be functional [57]. It must be noted, however, that mammalian (UV/blue) S-cones are sparse and have short telodendria, which makes it unlikely that they couple to each other either [93].

Recent progress on deciphering colour vision in invertebrates offers an interesting counterpoint to the problem of *heterochromatic* cone-cone coupling in vertebrates. Fruitfly microvillar photoreceptors specialise either in motion (photoreceptors R1–R6) or in colour discrimination (R7 and R8). The latter are functionally coupled to the former via gap junctions, with the effect of broadening the spectral tuning of motion discrimination [132]. On the other hand, the only direct interactions between the spectrally distinct R7 and R8 consist in reciprocal inhibition mediated by neurotransmitter release at their axon terminals [108]. Therefore, it appears that interphotoreceptor electrical coupling in the fruitfly is restricted to a pathway that is non critical for colour vision.

Similarly to *heterochromatic* cone-cone coupling, the existence of rod-cone coupling is certainly perplexing considering its potential degradation of chromatic information up to the mesopic-photopic transition, when rods begin to saturate (see discussion in [36]). The effect is exemplified by mouse cones, the vast majority of which coexpress M- and S-opsin in variable proportions: when a cone is strongly coupled to rods, its intrinsic spectral identity can only be uncovered by delivering a rod-saturating preflash (Fig. 3d) [5, 6]. One possibility is that strong rod-cone coupling may be used in species with a single cone spectral type to generate a third synthetic photoreceptor with mixed properties, thereby providing for improved colour discrimination. This idea emerged from the observation of a bimodal distribution in the amount of rod-cone coupling in the salamander retina, when at the time it was thought to have cones of a single spectral type [136]. One better candidate for this mechanism may be the northern hemisphere lamprey *L. fluviatilis*, whose rod-like and cone-like photoreceptors have absorbance peaks at 515 nm and 555 nm, respectively. Indeed, many lamprey cones—but not all—display significant rod input (Fig. 3a–c) [7], and there is evidence that this retina may be capable of colour discrimination in mesopic conditions [50]. It is not clear, however, why rods and cones shouldn’t be able to provide for better colour discrimination when uncoupled from each other, unless all direct communication between rod-like photoreceptors and bipolar cells has high gain and saturates with background light, as thought to occur in other lower vertebrates (see a preceding section). In fact, recent studies found that mouse rods contribute to colour discrimination through pathways different from rod-cone gap junctions [62, 66].

A second hypothesis is that rods couple preferentially to cones with similar or red-shifted action spectra (i.e. M/L-cones), while avoiding the blue (S-ones). The rationale for this is that due to the prominent secondary hypsochromic (i.e. blue-shifted) peak in the absorption spectrum of all opsins (the β-band [49]), rod input would have a limited impact on coupled cones anyhow. There is some very preliminary electrophysiological evidence in support of this, both in macaque [56] and mouse [5]. However, anatomically rods do form gap junctions with S-cones, at least in the peripheral human [69] and macaque retina [93]. In several amphibians two types of rods are present: a more numerous population of standard green-sensitive (GS) rods and a sparse population of blue-sensitive (BS) transmuted cones expressing S-opsin. In toad and frog, these photoreceptors have been shown, behaviourally, to confer to the animal colour discrimination in the scotopic range [139]. Thus, they represent a unique opportunity to examine the presence and impact of heterochromatic rod-rod coupling on colour discrimination. The data are unfortunately scant, with salamander BS rods only known to form telodendrial contacts with nearby BS partners, as well as with S-opsin expressing ‘proper’ cones [141]. It remains to be seen whether these rods receive electrical input from the more numerous (and strongly coupled) GS rods [45].

## Interphotoreceptor coupling varies greatly across species

There is a great variability across species in the relative extent of cone-cone, rod-rod, and rod-cone coupling. Among lower vertebrates, no evidence of cone-cone coupling has been found in salamander, either anatomically [33, 86] or electrophysiologically [12], while turtle cones are extensively coupled to each other [14, 36]. In mammals, cone-cone coupling appears to be a general trait (with the notable exception of S-cones) having been detected anatomically in cat [68], human [1], macaque [93, 125], mouse [126, 128] and confirmed electrophysiologically in squirrel [38], macaque [57] and mouse [61]. Considering that gains outweigh costs in cone-cone coupling, it would be surprising if it wasn’t the norm among vertebrates. In fact, the existence of species that don’t exploit such coupling is puzzling. A consistent feature among lower vertebrates, at least those in which electrophysiological recordings have been performed, is the extreme electrotonic pooling of signals among neighbouring rods via gap junctions. In contrast, mammalian rods operate largely independently of each other, although the exact degree is still debated. We discussed the important implications of these differences for visual processing in a previous section.

With regard to rod-cone coupling, based on the number of junctional particles, toad and frog rods appear much more strongly coupled to each other than to cones [48, 70]. Electrophysiological data confirmed this asymmetry in salamander [10, 12, 45] and turtle [32]. On the other hand mammalian rods tend to show, if any, a preference for coupling to cones over other rods [61, 68, 93, 100, 119, 126]. These stark differences are likely to be, in large part, the byproduct of a major reduction in the degree of rod-rod coupling in mammals. Rod-cone coupling has now been confirmed electrophysiologically in a wide variety of species (see ‘Providing an escape route for rod or cone signals’) and reconstructions of junctional contacts in the cat, mouse and peripheral macaque retina estimate a similar degree of convergence onto each cone (25–50:1 [93, 119, 126]). In summary, while differences in rod-rod coupling seem to be systematic and, as we have discussed, related to the evolution of specialised night vision circuitry in mammals, it is difficult to assess whether those in cone-cone and rod-cone coupling are representative of vertebrates or specific to the animal models used in vision research.

## Light and circadian modulation

Following a theoretical prediction that cat rods and cones decouple in darkness to prevent shunting of the rod single photon response [119], light has indeed been found to promote rod-cone coupling in salamander [138] and frog [70]. In stark contrast, bright light causes the opposite effect in goldfish, an uncoupling of rods and cones [102]. Even more surprisingly, these opposite effects have been linked to the same modulatory pathway: the well-known light-evoked increase in the retinal release of dopamine (DA) would act via D2-like receptors expressed on photoreceptors, thereby modifying the phosphorylation state of Cx35/36 [79]. Of note, a specular role for adenosine has also been proposed [80]. In mouse, changes in tracer diffusion upon cut loading [80, 102] and indirect electrophysiological evidence [55] suggest that rod-cone coupling is strong in darkness and suppressed by light, similarly to what occurs in goldfish and contrary to the theoretical expectations of Smith and colleagues [119]. However, no modulatory effects of light or DA on rod-cone coupling have been found to date in macaque [110]. Whether rod-rod and cone-cone coupling are subject to analogous modulatory changes is not clear. For instance, while in frog DA promotes rod-cone coupling it has no effect on rod-rod coupling [70]. Also, DA seems not to modulate cone-cone coupling in squirrel [38, 83] and indirect psychophysical evidence suggests that light does not affect cone-cone coupling in human [38]. In addition to the above acute effects of light, circadian rhythms in neuromodulator release have been found to alter rod-cone coupling in several species. In goldfish, zebrafish and mouse, this type of coupling is strong at night but weak during the day, even after long dark adaptation [79, 102]. In contrast, no evidence of circadian rhythmicity has been found in salamander [45, 138]. Considering that many studies have reported a large variability in interphotoreceptor coupling in the same retina [5, 7, 38, 56, 57, 70, 74, 110, 136], one may wonder to what extent this is due to intrinsic heterogeneity or to differences in modulatory state.

Note that in this review we do not touch on the important topics of developmental or lesion-induced changes in connexin expression and electrical coupling.

## Open issues and hypotheses

### The perceptual impact of coupling

In spite of the clear potential benefits of interphotoreceptor coupling (Fig. 2) and its widespread presence in vertebrates, the actual impact it has on visual perception remains largely unproven. Even in mammals, where a large research community using transgenic and psychophysical approaches should help to shine light on this matter, unambiguous answers are lacking. For instance, cone-cone gap junctions are very well documented in the peripheral retina of macaque [57, 93, 125], but nearby cones do not display much functional correlation or direct interaction in the way they influence ganglion cell activity [44, 81]. Also, difficult to reconcile is the indiscriminate coupling between primate M- and L-cones with its potential degradation of crucial chromatic information on green–red hues (e.g. fruit ripeness). Similarly, the exact extent of mammalian rod-rod and rod-cone coupling and how they affect retinal processing of single photon responses at absolute visual threshold remain to be quantified.

As for rod-cone coupling, while great efforts have been made to dissect its contribution in mammals, and psychophysical correlates of this secondary rod pathway have been suggested (reviewed in [52]), it is now clear that rod signals use a multiplicity of routes toward the inner retina, whose relative importance is only partly understood [41]. These routes rely on direct synapses of rods with a subpopulation of OFF cone bipolars ([15, 20, 126, 128] and references therein) and lateral inhibition on cones via horizontal cells [62]. In the mouse, there is also some evidence of direct rod input to a subset of ON cone bipolars ([20, 96, 127] but see [15]). Such redundancy of rod pathways greatly complicates the interpretation of psychophysical data and in vitro experiments. Additional confounding factors are (i) Cx36 is widely expressed throughout the retina; (ii) changes in rod-cone coupling have not only been linked to ambient light and circadian time, but occur in response to perturbation of the photoreceptors [4, 56]; (iii) coupling strength varies even between strains of the same animal species [61]. Recent evidence indicates that rod vision in mesopic conditions may be partly or wholly independent of rod-cone coupling in both macaque [52] and mouse [97]. Other striking observations in apparent conflict with a major role of rod-cone coupling are (i) the recent discovery of a class of mouse retinal ganglion cells that receive no detectable rod input in their receptive field centre [62]; (ii) the almost complete absence of ganglion cells whose activity is affected by light in scotopic conditions in a rod bipolar cell mouse mutant [115]. On the other hand, there is evidence that cone-mediated responses are progressively enhanced by the removal of shunting to rods via junctional decoupling during bright light adaptation [84]. In summary, much remains to be understood about the physiological role of interphotoreceptor coupling.

### What about radial fins?

A question that remains unanswered is whether electrical communication at radial fins is a common vertebrate trait. In the toad, rods mutually interconnect in a tight electrical syncytium, as well as to cones, via these processes [48], while the contribution of telodendria has been questioned. Similar observations have been made in another amphibian, the salamander [86]. Crucially, in this animal Cx35 expression colocalises with the radial fins [140], as well as the outer plexiform layer. Oddly, in frogs, gap junctions seem to occur slightly distally to the radial fins [70]. Thus, in at least some amphibian species, both radial fins and telodendria participate in coupling. However, in other vertebrate classes, such a role of fins has not emerged ([93, 95, 101, 104] but see [28] and references therein) and today they are seldom mentioned in the literature. Considering their widespread occurrence from lamprey to human, it is plausible that amphibians recruited for coupling these pre-existing structures, whose general function in vertebrates is still unknown. Another apparent peculiarity of amphibians is their lack of cone-cone coupling and opposite (relative to other species) regulatory effect of light on rod-cone coupling. A second possibility is that the role of radial fins in electrical coupling is currently under-appreciated due to the small sample of vertebrates examined to date. Perhaps the fins played a greater role in the coupling of ancestral photoreceptors, a hypothesis that may be worth examining in lampreys.

### An ancestral role for vesicular neurotransmitter release

The question of whether interphotoreceptor coupling also involves neurotransmitter release has been lingering in the literature for decades (e.g. [47, 51]), fuelled by sporadic evidence of synaptic ribbons, a key player in neurotransmitter release by photoreceptors [122], juxtaposed to the axon terminals or telodendria of neighbouring photoreceptors in salamander [33, 76], turtle [87, 95] and even human, where synaptic vesicles cluster at the junctional contact between S-cone pedicles and nearby cone telodendria [69]. Oddly, it appears that these types of contacts are restricted to *heterocellular* (i.e. rod-cone) and *heterochromatic* coupling. While mixed electrical/glutamatergic synapses are more widespread in vertebrates than previously appreciated [99], as far as we know no functional counterpart to the above mentioned anatomical observations has yet emerged: blocking chemical transmission in toad, turtle (references in [95]), squirrel [38] and macaque [57] had no effect on coupling, and despite careful inspection our group found no evidence whatsoever for residual rod-cone coupling in Cx36-KO mice [6]. Whatever the modern physiological role of these types of contacts, if any, it is possible that coupling in ancestral photoreceptors occurred via mixed synapses. Intriguingly, the ciliary photoreceptors in the ocellus of the chordate ascidian *Ciona intestinalis* larva, thought to be homologous to the ciliary retinal photoreceptors in vertebrates, mutually interconnect via chemical and electrical synapses [106]. A vertebrate model that could preserve evidence of such an ancestral role is the lamprey: (i) it retains archaic morphological features; (*ii*) interphotoreceptor coupling is strong [7]; (iii) photoreceptors possess synaptic ribbons near the outer limiting membrane [137]; (iv) hybrid synapses play an important role in its nervous system.

### Beyond image forming photoreceptors

As for other aspects of the visual system, it is likely that interphotoreceptor coupling already shaped photoreception before its widespread deployment in the vertebrate retina. Its most likely ancestral function would have been that of improving SNR by averaging uncorrelated noise, as it occurs in vertebrate cones, with the purpose of decreasing the threshold for phototactic and other light-dependent behaviours. This hypothesis might be tested in the sensory organs of basal chordates and in the vertebrate pineal gland, where ciliary photoreceptors are present. Relatively little is known about these systems, but the ascidian larva ocellar photoreceptors interconnect by mixed synapses [106] and the parapinopsin-expressing photoreceptors in the pineal gland of lamprey are coupled by gap junctions [64]. Unfortunately, no data are available for the frontal eye photoreceptors of the amphioxus.

Another intriguing possibility stems from the proposal that retinal bipolar cells diverged from ciliary photoreceptors in a chordate or early vertebrate ancestor [73, 89]: the two classes of retinal neurons could have retained some common features in their respective molecular components and regulatory machinery for homocellular communication. Notably, cone bipolars in rabbit were recently found to establish extraordinarily extensive, yet specific, mutual gap junctional coupling [117]. Coupling between bipolar cells may thus be more prevalent in vertebrates than previously thought.

Ciliary photoreceptors diversified through the complex interplay of gene duplications/deletions, developmental recruitment/derecruitment events, transmutations, shaped in the medium term by changes in ecological niche and in the long term by the progressive sophistication of the visual system [29]. Interphotoreceptor coupling, likely already present in our last common ancestor with lampreys in the Cambrian period, accompanied this evolutionary history while being fine-tuned in response to a changing balance of pressures for or against its expression. Highly relevant for us, yet largely unexplored, is the transition from the tightly coupled rod network of lower vertebrates to the patchy one of mammals. In fact, several major questions on the evolution and physiological role of coupling remain to be adequately answered, which makes this classical topic of visual neurophysiology still interesting and deserving of future investigation.
